# Supplementary material for: Two dominant boreal conifers use contrasting mechanisms to reactivate photosynthesis in the spring
Source: Nat Commun. 2020 Jan 8;11:128. doi: 10.1038/s41467-019-13954-0 (PMC6949249; doi:10.1038/s41467-019-13954-0)
Supplement: Supplementary file 1 — Supplementary Information [file 41467_2019_13954_MOESM1_ESM.pdf]

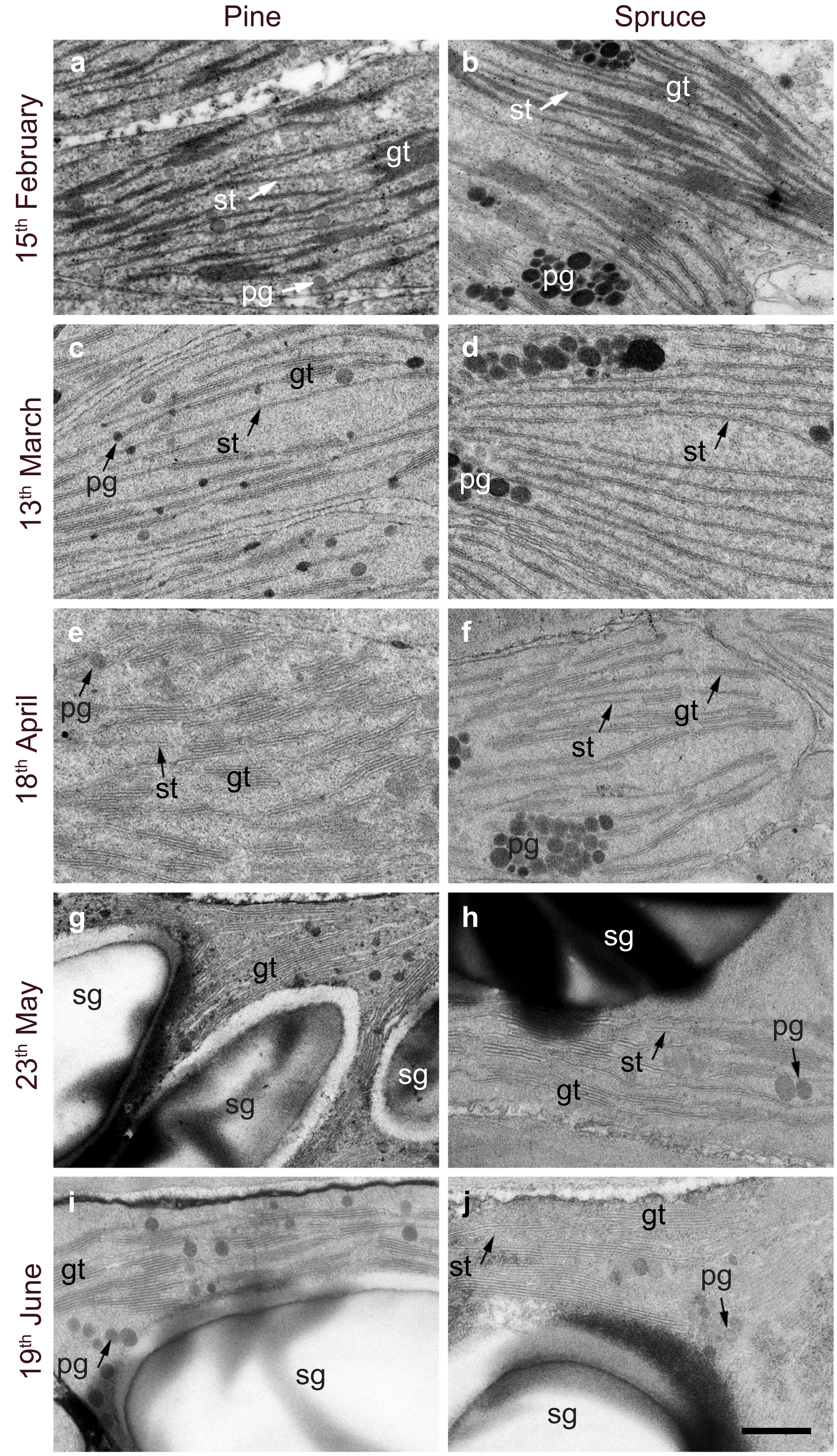

Supplementary Figure 1. Transmission electron micrographs (TEM) of chloroplast structures in Scots pine and Norway spruce. Needles were collected in field during **(a, b)** February, **(c, d)** March, **(e, f)** April, **(g, h)** May and **(i, j)** June. Representative images are shown. Bar: 0.5um. gt, grana thylakoid (stacked); st, stromal thylakoid (unstacked); sg, starch granule; pg, plastoglobulus.

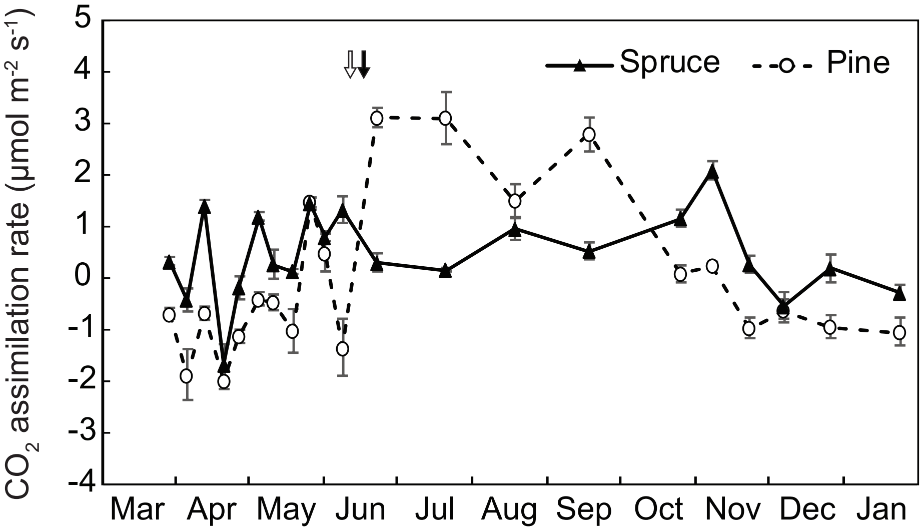

Supplementary Figure 2. Seasonal photosynthetic CO_2 assimilation rate in Scots pine and Norway spruce. Measurements were performed under ambient conditions (light intensity, 500 µmol photons m^{-2} s^{-1} and CO_2 concentration, 400 µmol m^{-2} s^{-1}, 23 °C). Samples were collected from March 2017 through January 2018. The dates of budburst in Scots Pine and Norway spruce are indicated with open and closed arrows, respectively. Each data represents mean of 6 independent samples (± SE).

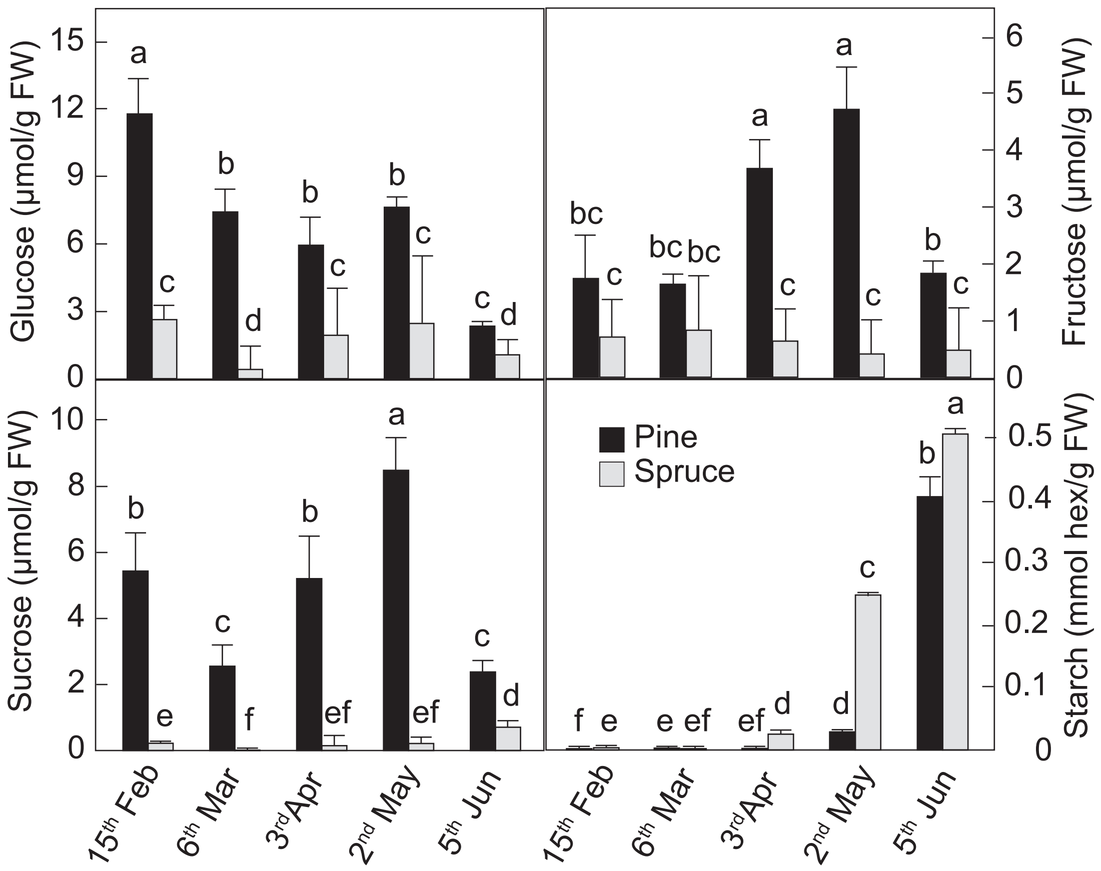

Supplementary Figure 3. Soluble carbohydrates and starch in Scots pine and Norway spruce needles during the winter to summer transition period. The samples were collected in the morning. Significant differences were indicated with different letters above the bars (One-way ANOVA, $P<0.05$). Each bar shows the mean of 3 independent samples (± SD).

a

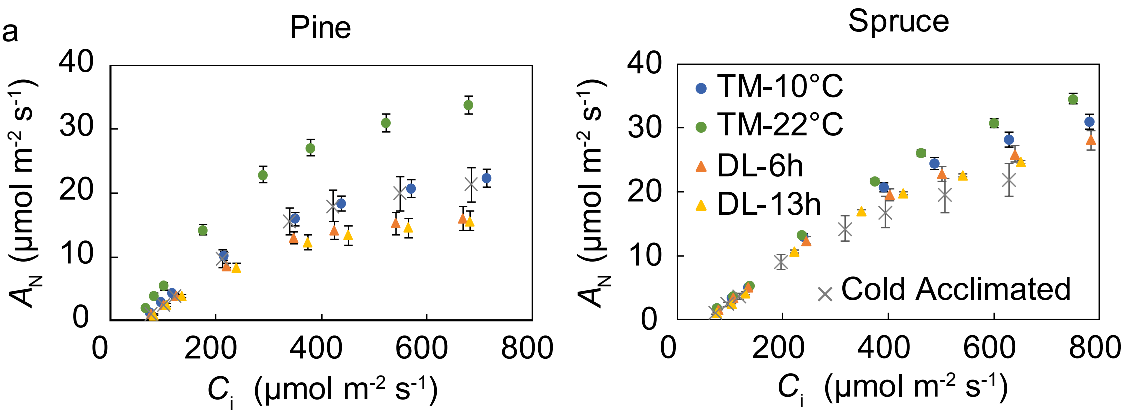

b

Significance		A_N	V_{cmax}	J_{max}
Cold vs. Warm	pine	***	**	***
	spruce	ns	ns	**
SD vs. LD	pine	ns	ns	ns
	spruce	ns	ns	ns

Supplementary Figure 4. The responses of CO_2 assimilation capacity to temperature and day length variation in Scots pine and Norway spruce seedlings. (**a**) Response of assimilation (A_N) to the internal CO_2 concentration (C_i) in seedlings grown in climate chambers. The A/C_i curves were measured in one-year-old cold acclimated Scots pine and Norway spruce seedlings (indicated with ×). Seedlings were transferred either to temperature (TM) controlled chamber (temperature was increased from 4 to 22°C by 1°C per day, with 8/16 h light/dark cycle) or day length (DL) controlled chamber (day length was increased from 4 to 22h by 1h per 2 days, under 5°C) and A/C_i curves were determined two more time point after the transfer. Each data represents mean of 4-6 independent samples (± SE). (**b**) Effects of temperature and day length on the photosynthetic parameters in Scots pine and Norway spruce seedlings. A_N, net CO_2 assimilation rate; V_{cmax}, maximum rate of carboxylation; J_{max}, maximum rate of electron transport. Parameters were calculated from the A/C_i curve showed in (**a**) and Fig. 3c. Two-tail T-Test indicates statistically significant difference caused by cold and warm temperature between Pine-C with DL-13h and Pine-W with TM-22°C. And the significant difference caused by short day (SD) and long day (LD) were analyzed between Pine-C with TM-22°C and Pine-W with DL-13h. **, $P < 0.01$; ***, $P < 0.001$; ns, not significant.

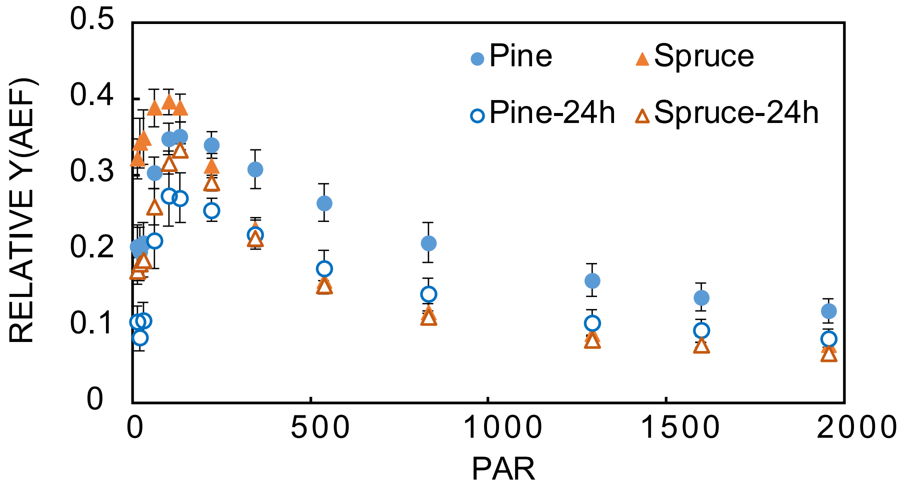

Supplementary Figure 5. Alternative electron flows (AEF) changes in Scots pine and Norway spruce spring samples in a controlled recovery experiment. Relative yield of AEF, Y(AEF), is calculated as Y(AEF) = Y(I) – Y(II). The yield of PSI and PSII were measured simultaneously with rapid light curves. Samples of Scots pine (circle) and Norway spruce (triangle) were collected from the field in April, and measured immediately (closed symbols) and after recovered in room temperature for 24 hours (open symbols). Each data point represents the mean of 4 independent samples (± SE).

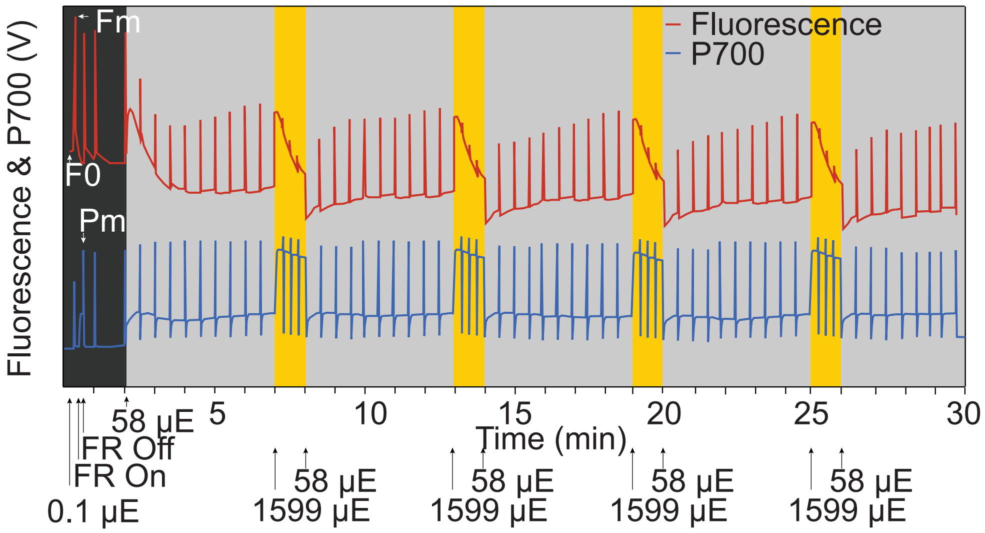

Supplementary Figure 6. Photosynthetic fluorescence measurement of Scots pine and Norway spruce under illumination mimicking the fluctuating light conditions in the field. The example of fluorescence (red line) and P700 (blue line) curves monitored with 2 min dark, followed by four cycles of 5 min low light (58 µmol photons m^{-2} s^{-1}) and 1 min high light (1599 µmol photons m^{-2} s^{-1}).

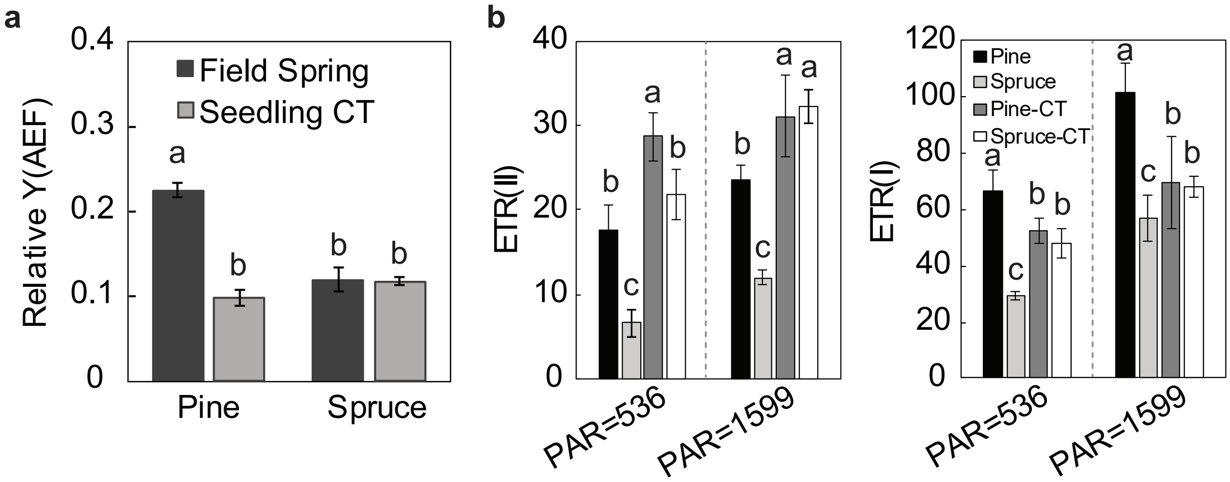

Supplementary Figure 7. Chlorophyll fluorescence parameters of Scots pine and Norway spruce under high light conditions during steady state of photosynthesis. (a), Relative yield of AEF (Y(AEF)). Y(AEF) is calculated as Y(AEF) = Y(I) – Y(II). The yield of PSII and PSI were measured simultaneously under moderate high light (536 µmol photons m^{-2} s^{-1}). **(b),** ETR(II), electron transport rate through PSII. ETR(I), electron transport rate through PSI. Light intensity was 536 µmol photons m^{-2} s^{-1} or 1599 µmol photons m^{-2} s^{-1}. Samples of pine (black) and spruce (light gray) were collected from field in April. Samples of pine (dark gray) and spruce (white) seedlings grown in growth chamber (22 °C, 150 µmol photons m^{-2} s^{-1} and 8/16h light/dark cycle) were used as control (CT). Significant differences were indicated with different letters above the bars (One-way ANOVA, $P<0.05$). Each bar shows the mean of 4 independent samples (± SD).

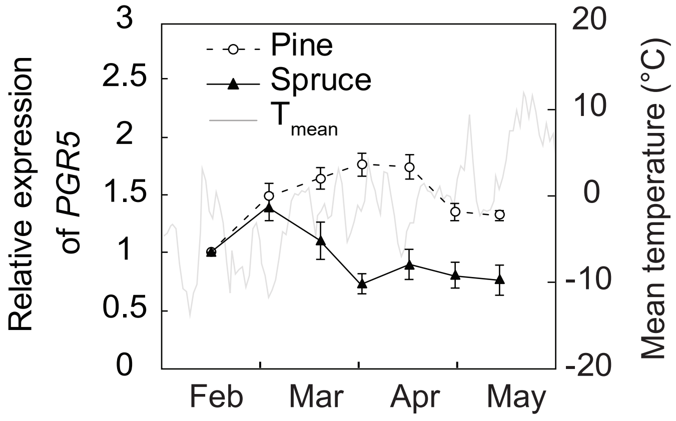

Supplementary Figure 8. *PGR5* expression during the spring recovery phase in Scots pine and Norway spruce. Relative expression values were normalized against the reference gene *PP2A* and related to the amount present in the February samples. Each data point represents the mean (±SE) of at least 3 independent replicates. The daily mean of air temperature for the period February to May 2017 is shown in light grey.

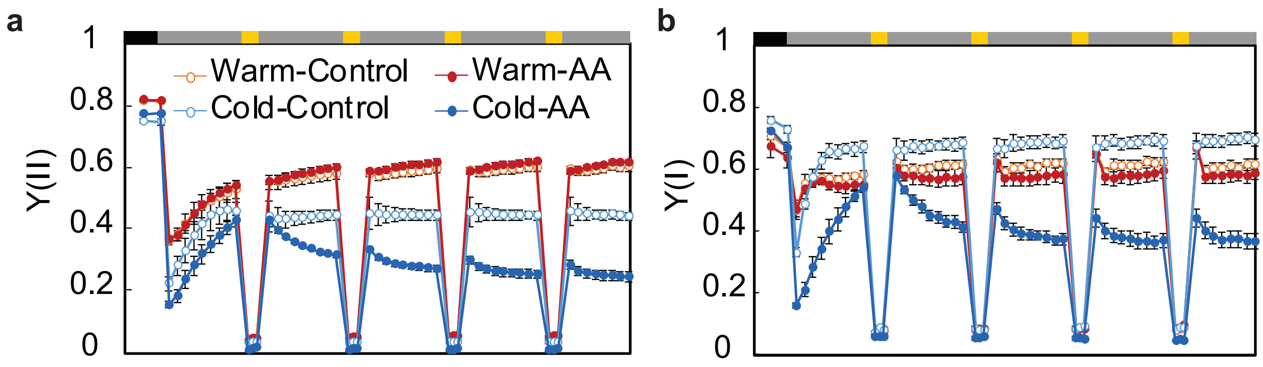

Supplementary Figure 9. Effect of antimycin A (AA) treatment upon light fluctuations in Scots pine seedlings grown in cold and in warm control conditions. The parameters Y(II) in (**a**), operating efficiency of PSII, and Y(I) in (**b**), operating efficiency of PSI are shown. Needles were collected from cold acclimated pine seedlings grown under cold (5 °C, indicated with light and dark blue circles) or warm (22 °C, indicated with orange and red circles) conditions for three weeks. Needles were treated with water (open circles) or 200 µmol AA (closed circles). The same protocol applied in Fig. 4c-h was used for the measurements. Each data point represents the mean of 4 independent samples (±SE).

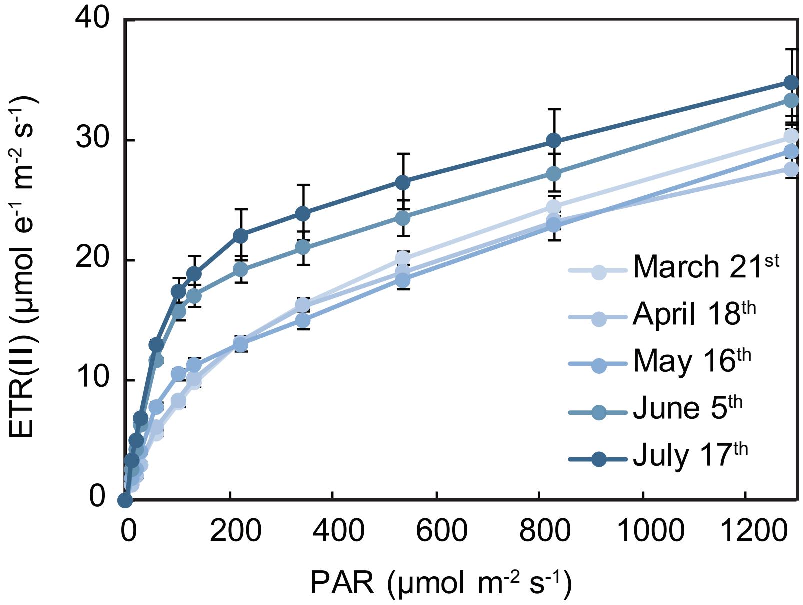

Supplementary Figure 10. ETR(II) in Scots pine were higher in summer than spring via light curve measurement. ETR(II), the electron transport rate of PSII. Samples were collected from March to July 2017. Each data represents mean of 6 independent samples (± SE).

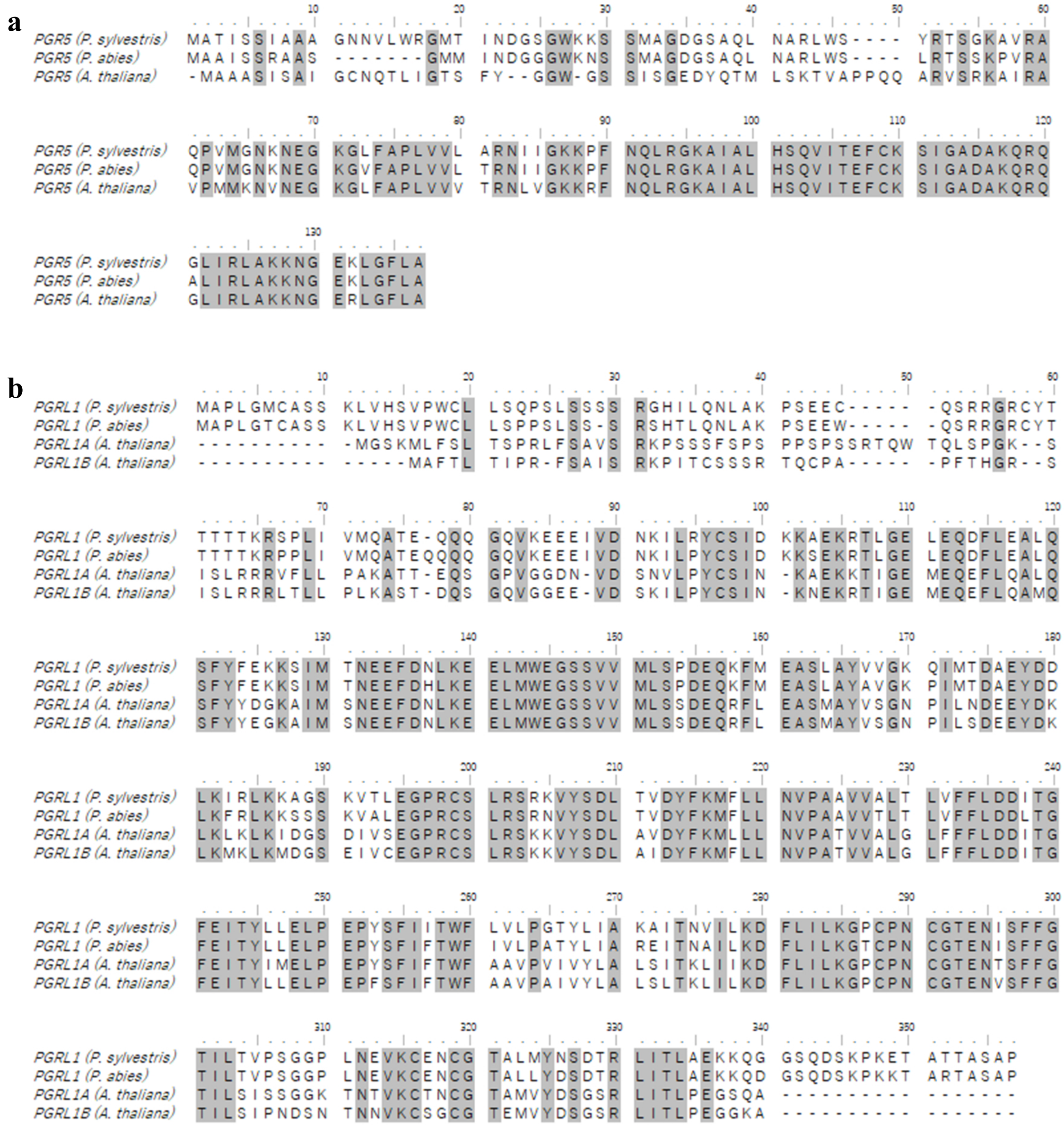

Supplementary Figure 11. Sequence alignments for PGR5 and PGRL1 proteins. Alignments for PGR5 (**a**) and PGRL1 (**b**) protein sequences from *Pinus sylvestris*, *Picea abies* and *Arabidopsis thaliana* are presented. Conserved amino acids are shaded in gray.

Supplementary Table 1. Primers used for Real-time PCR.

Species	Gene	Primer Name	Primer Sequence 5'-3'
Pinus sylvestris	*PGR5*	PsPGR5-F2	TGGTGCTCGCTCGTAATATC
		PsPGR5-R2	TGTCTTTGTTTGGCATCAGC
	Rbcl	PsRBCL-F2	TTTGGAAGATTTGCGGATTC
		PsRBCL-R2	GAGTCCACCACGGAGACATT
	PP2A	PsPP2A-F1	CAAGCCGACATCATGACCTG
		PsPP2A-R1	GGACAGGGTTCCCAACATCA
Picea abies	*PGR5*	PaPGR5-F2	CAACCAATTGAGAGGAAAAGC
		PaPGR5-R2	CTTAGCCAAGCGAATCAAGG
	Rbcl	PaRBCL-F1	CGTTGCTGGAGAGGAAAGTC
		PaRBCL-R1	GAATCCGCAAATCTTCCAAA
	PP2A	PaPP2A-F1	GCTGCTGGAAAGGTGACAAA
		PaPP2A-R1	CAATTGTTGCCTCCTTCCCC
