# Supplementary material for: Two dominant boreal conifers use contrasting mechanisms to reactivate photosynthesis in the spring
Source: Nat Commun. 2020 Jan 8;11:128. doi: 10.1038/s41467-019-13954-0 (PMC6949249; doi:10.1038/s41467-019-13954-0)
Supplement: Supplementary file 3 — Reporting Summary [file 41467_2019_13954_MOESM3_ESM.pdf]

nature research

Corresponding author(s): Åsa Strand, NCOMMS-19-27330A

Last updated by author(s): Nov 29, 2019

Reporting Summary

Nature Research wishes to improve the reproducibility of the work that we publish. This form provides structure for consistency and transparency in reporting. For further information on Nature Research policies, see Authors & Referees and the Editorial Policy Checklist .

Statistics

For all statistical analyses, confirm that the following items are present in the figure legend, table legend, main text, or Methods section.

n/a	Confirmed	
☐	☒	The exact sample size (n) for each experimental group/condition, given as a discrete number and unit of measurement
☐	☒	A statement on whether measurements were taken from distinct samples or whether the same sample was measured repeatedly
☐	☒	The statistical test(s) used AND whether they are one- or two-sided<br>*Only common tests should be described solely by name; describe more complex techniques in the Methods section.*
☒	☐	A description of all covariates tested
☒	☐	A description of any assumptions or corrections, such as tests of normality and adjustment for multiple comparisons
☐	☒	A full description of the statistical parameters including central tendency (e.g. means) or other basic estimates (e.g. regression coefficient) AND variation (e.g. standard deviation) or associated estimates of uncertainty (e.g. confidence intervals)
☐	☒	For null hypothesis testing, the test statistic (e.g. F, t, r) with confidence intervals, effect sizes, degrees of freedom and P value noted<br>*Give P values as exact values whenever suitable.*
☒	☐	For Bayesian analysis, information on the choice of priors and Markov chain Monte Carlo settings
☒	☐	For hierarchical and complex designs, identification of the appropriate level for tests and full reporting of outcomes
☒	☐	Estimates of effect sizes (e.g. Cohen's d, Pearson's r), indicating how they were calculated

Our web collection on statistics for biologists contains articles on many of the points above.

Software and code

Policy information about availability of computer code

Data collection	No software was used.
Data analysis	We used ImageJ V.1.51j8 and Microsoft Excel 365 to analyze the data.

For manuscripts utilizing custom algorithms or software that are central to the research but not yet described in published literature, software must be made available to editors/reviewers. We strongly encourage code deposition in a community repository (e.g. GitHub). See the Nature Research guidelines for submitting code & software for further information.

Data

Policy information about availability of data

All manuscripts must include a data availability statement. This statement should provide the following information, where applicable:
- Accession codes, unique identifiers, or web links for publicly available datasets
- A list of figures that have associated raw data
- A description of any restrictions on data availability

The underlying Figs 1, 2b–d, 3, 4 and 5 and Supplementary Figs 2, 3, 4a, 5, 7, 8, 9 and 10 are provided as a Source Data file. Any other data that support the findings of this study are available within the manuscript and its supplementary files or are available from the corresponding author(s) upon request.

Field-specific reporting

Please select the one below that is the best fit for your research. If you are not sure, read the appropriate sections before making your selection.

☒ Life sciences ☐ Behavioural & social sciences ☐ Ecological, evolutionary & environmental sciences

Life sciences study design

All studies must disclose on these points even when the disclosure is negative.

Sample size	Sample size varied from n=4 to n=6, providing sufficient replication to calculate mean and SE. Western blots (Fig 5) were performed in triplicate to provide sufficient replication for accurate determination of abundance and extraction efficiency.
Data exclusions	No data was excluded and all data is provided in the Source data file.
Replication	Experimental data was collected with replication as described in the sample size section above.
Randomization	Samples for measurements were collected at random from available populations of branches or seedlings as appropriate for the experiment.
Blinding	Blinding was not relevant to these experiments.

Reporting for specific materials, systems and methods

We require information from authors about some types of materials, experimental systems and methods used in many studies. Here, indicate whether each material, system or method listed is relevant to your study. If you are not sure if a list item applies to your research, read the appropriate section before selecting a response.

Materials & experimental systems

n/a	Involved in the study	
☐	☒	Antibodies
☒	☐	Eukaryotic cell lines
☒	☐	Palaeontology
☒	☐	Animals and other organisms
☒	☐	Human research participants
☒	☐	Clinical data

Methods

n/a	Involved in the study	
☒	☐	ChIP-seq
☒	☐	Flow cytometry
☒	☐	MRI-based neuroimaging

Antibodies

Antibodies used	Antibodies were raised against the PGR5 and PGRL1 of Arabidopsis (Agrisera, catalog number: AS16 3985 and AS19 4311) and antibody of FLVB was provided by Dr. Toshiharu Shikanai (Kyoto University).
Validation	The three antibodies used in our manuscript for Western Blot were validated in Scots pine and Norway spruce by us. For PGR5 and PGRL1 antibodies, one can find relevant validation information on the manufacturer's website.
